# Dendritic Mesoporous Silica Nanoparticle-Based Co-Delivery of a Repellent and an Insecticide for Management of the Asian Citrus Psyllid

**DOI:** 10.3390/nano16191206

**Published:** 2026-09-24

**Authors:** Wenhua Rao, Qianying Li, Kaidi Wang, Hua Chen, Lan Li, Chao Shen, Guocheng Fan, Jinfeng Hu

**Affiliations:** 1 Fujian Engineering Research Center for Green Pest Management, Fujian Key Laboratory for Monitoring and Integrated Management of Crop Pests, Fuzhou Scientific Observing and Experimental Station of Crop Pests of Ministry of Agriculture and Rural Affairs, Institute of Plant Protection, Fujian Academy of Agricultural Sciences, Fuzhou 350013, China; 2 College of Plant Protection, Fujian Agriculture and Forestry University, Fuzhou 350002, China; 3 Ningde Inspection and Testing Centre for Agricultural Product Quality and Safety, Ningde 352100, China; 4 Fujian Provincial Center for Agri-Product Quality Safety Inspection and Testing, Fuzhou 350003, China

**Keywords:** SiO_2_, *Diaphorina citri*, Huanglongbing, fir essential oil, imidacloprid

## Abstract

The Asian citrus psyllid (*Diaphorina citri*) serves as the principal insect vector responsible for the transmission of citrus Huanglongbing (HLB). Consequently, the effective control of *D. citri* is of paramount importance for curbing the spread of this devastating disease. Fir essential oil (FEO) exhibits a potent repellent activity against *D. citri*; however, its practical application is severely constrained by a short duration of efficacy arising from its high volatility. Imidacloprid (Im) is a conventional insecticide widely used for *D. citri* management, and prolonging its residual activity remains a critical challenge for field application. In this study, we synthesized dendritic mesoporous silica nanoparticles (DMSNs, ≈257 nm) and employed them to construct a nano-pesticide formulation co-loaded with both Im and FEO. Our results demonstrated that DMSNs possessed high loading capacities for Im (21.79%) and FEO (32.98%). Notably, both Im and FEO within the DMSNs-Im-FEO formulation exhibited favorable sustained-release profiles and significantly extended residual activities compared to their free counterparts. Furthermore, the DMSNs-Im-FEO formulation displayed preliminary non-target tolerance in citrus plants, Chinese milk vetch, and earthworms, indicating its low ecotoxicological risk. Overall, our study provides a novel and promising strategy for the sustainable management of *D. citri*, which holds considerable significance for the integrated management of HLB.

## 1. Introduction

The devastating prevalence of citrus Huanglongbing (HLB) represents a major factor constraining the sustainable development of the global citrus industry [1]. In Florida alone, the disease has reduced annual citrus production by approximately 80% [2]. Given the absence of any curative or effective therapeutic measure to date [3], HLB is often colloquially referred to as “citrus cancer.” Currently, the most effective management strategy remains the integrated “three-pronged” approach developed and widely promoted in China, which combines the use of pathogen-free nursery stock, the prompt removal of infected trees, and the intensive suppression of the Asian citrus psyllid (*Diaphorina citri*) [2,4]. This psyllid serves as the primary vector of *Candidatus* Liberibacter asiaticus (*C*Las), the causal agent of HLB [5,6]. Notably, once psyllids acquire *C*Las from infected trees, they exhibit enhanced fecundity and flight capacity [7,8], accompanied by a stronger preference for healthy citrus hosts [9]—all of which synergistically accelerate pathogen dissemination. Consequently, timely and effective vector control is of critical importance for interrupting the HLB transmission cycle.

Chemical insecticides, such as imidacloprid (Im), currently constitute the cornerstone of psyllid management programs. In practical application, however, their residual activity is frequently suboptimal, and the escalating prevalence of insecticide resistance [10,11,12] compels growers to apply increasingly higher dosages, thereby giving rise to serious environmental concerns [13,14]. Therefore, extending the effective duration of commonly used insecticides like Im represents an urgent and pressing challenge. Advances in nanotechnology have opened up new avenues for addressing this challenge [15,16,17]. Among the various nanocarriers explored to date, mesoporous silica nanoparticles (MSNs) have attracted particular attention owing to their large specific surface area, excellent biocompatibility, and highly tunable structural properties, which enable enhanced pesticide retention on target foliage, improved photostability, and prolonged residual efficacy [18,19,20,21,22,23]. Dendritic mesoporous silica nanoparticles (DMSNs), a distinctive subclass of MSNs, feature a three-dimensional center-radial pore architecture that affords exceptionally high pore volume and superior active-site accessibility, thereby offering clear advantages for high-capacity loading and sustained release of active ingredients [24,25,26]. Accordingly, the development of DMSNs-based nano-pesticide formulations for psyllid control holds considerable promise.

Meanwhile, plant-derived repellents have garnered increasing interest for integrated psyllid management, largely owing to their favorable environmental profiles [27,28,29,30]. Fir essential oil (FEO) has been demonstrated to possess potent repellent activity against *D. citri* [29]. However, like many essential oils, its high volatility drastically curtails its effective lifespan under field conditions, severely limiting its practical deployability [31,32]. Encouragingly, encapsulation of essential oils within DMSNs has emerged as a proven strategy to substantially extend their persistence [24,33,34,35]. Hence, the development of a DMSNs-based long-lasting FEO repellent formulation could contribute meaningfully to reducing HLB incidence in citrus orchards.

In the field context, effective psyllid management demands not only rapid knockdown to suppress population build-up but also sustained repellency to minimize direct contact between psyllids and citrus trees, thereby reducing the risk of pathogen transmission [2,5]. Studies have demonstrated that the combination of insecticides and repellents can improve the management of insect vectors responsible for transmitting animal pathogens [36]. However, to the best of our knowledge, no study has yet reported the co-encapsulation of a synthetic insecticide and a plant essential oil within a single nanocarrier platform to construct a dual-functional formulation that simultaneously delivers both insecticidal and repellent actions. How to successfully co-load two active ingredients with disparate physicochemical properties and enable their combined repellent and insecticidal functions over an extended period under field conditions remains a fundamental question.

In the present study, we employed DMSNs as a nanocarrier platform to co-load Im and FEO, thereby fabricating a dual-functional nano-formulation. We systematically evaluated its co-loading performance, the sustained-release profiles of both active ingredients, and its repellent and insecticidal efficacy against *D. citri*, together with a preliminary biosafety assessment (Scheme 1). The overarching goal of this work is to provide a novel strategy for psyllid control and to offer technical support for disrupting the HLB transmission cycle, thereby contributing to the sustainable management of this devastating citrus disease.

## 2. Materials and Methods

### 2.1. Materials

Cetyltrimethylammonium bromide (CTAB), tetraethyl orthosilicate (TEOS), and sodium salicylate (NaSal) were procured from Macklin (Shanghai, China). Triethanolamine (TEA) was obtained from Aladdin (Shanghai, China). Ethanol and methanol were purchased from Xilong Scientific (Guangzhou, China). Hydrochloric acid (HCl) was acquired from Sinopharm Chemical Reagent (Shanghai, China). Fir essential oil (FEO) was supplied by Guangzhou Ri Hua Flavor & Fragrance Co. (Guangzhou, China). All other reagents were of analytical grade and were used as received without further purification. Ultrapure water (Direct-Q 3, Merck Millipore, Billerica, MA, USA, 18 MΩ·cm) was employed throughout all experiments.

### 2.2. Rearing of D. citri

Healthy adult *D. citri* individuals were collected from *Murraya paniculata* plants growing in a public park in Fuzhou, China. The collected psyllids were subsequently reared indoors on potted *M. paniculata* seedlings bearing young shoots, under controlled environmental conditions (26 °C, 14 h light: 10 h dark photoperiod), without exposure to any pesticides.

### 2.3. Synthesis and Characterization of DMSNs

DMSNs were synthesized following a previously reported method [37] with minor modifications. Briefly, TEA (0.136 g) was dissolved in 50 mL of ultrapure water, and the solution was gently stirred (~300 rpm) in an oil bath (DF-101S, Yuhua, Gongyi, Henan, China) preheated to 80 °C. After 0.5 h, CTAB (0.76 g) and NaSal (0.336 g) were sequentially added, and stirring was continued for an additional 1 h. Subsequently, 8 mL of TEOS was added dropwise via a constant-pressure dropping funnel. Upon completion of the addition, the reaction mixture was maintained at 80 °C for a further 2 h. The resulting product was collected by centrifugation at 12,000 rpm for 15 min and washed three times with ethanol and water. To remove the CTAB template, the obtained material was dispersed in 240 mL of methanol containing 3.08 mL of HCl and refluxed at 60 °C for three cycles, each lasting 6 h. After vacuum drying at room temperature overnight (CV200, Jiaimu, Beijing, China), the DMSNs were obtained as a white powder.

The morphology and structure of the DMSNs were characterized by scanning electron microscopy (SEM; SU8010, Hitachi, Tokyo, Japan) and transmission electron microscopy (TEM; JEM-2100F, JEOL, Akishima, Tokyo, Japan). For SEM imaging, the samples were sputter-coated with gold to enhance conductivity, and the images were acquired at an acceleration voltage of 5 kV. For TEM observation, a small amount of sample was dispersed in water by ultrasonication for 10 min, and then a drop of the suspension was deposited onto a carbon-coated copper grid and air-dried; the TEM images were obtained at an accelerating voltage of 120 kV. Surface functional groups were analyzed by Fourier-transform infrared spectroscopy (FT-IR; IS10, Thermo Scientific, Waltham, MA, USA) in attenuated total reflectance (ATR) mode over the wavenumber range of 4000–400 cm^−1^ with a resolution of 4 cm^−1^ and 32 scans. The specific surface area was determined using a surface area analyzer (ASAP 2000, Micromeritics, Norcross, GA, USA) with N_2_ adsorption–desorption isotherms recorded at 77 K. Samples were degassed at 25 °C for 6 h under vacuum prior to measurement. The specific surface area was calculated by the Brunauer–Emmett–Teller (BET) method, and the pore size distribution was derived by the Barrett–Joyner–Halenda (BJH) method.

### 2.4. Preparation of DMSNs-Im-FEO

Im was first loaded onto DMSNs using a solvent evaporation method [38]. In a fume hood, 250 mg of Im was weighed into a round-bottom flask and dissolved in 50 mL of methanol under sonication. Subsequently, 250 mg of DMSNs was added, and the mixture was sonicated to achieve uniform dispersion. The flask was then placed in a water bath at 60 °C and stirred under sealed conditions for 4 h. Thereafter, the cap was removed, and stirring was continued until the methanol had nearly evaporated, leaving the solids in a moist state. To redisperse the product, 10 mL of ethanol was added, and the mixture was centrifuged at 12,000 rpm for 5 min. After removal of the supernatant, the product was vacuum-dried at room temperature to yield DMSNs-Im. The Im loading content in DMSNs-Im was determined by UV spectrophotometry [39] (T10, Purkinje, Beijing, China) (Text S1 and Figure S1, Supporting Information).

Essential oil loading was performed following a previously reported method [33]. In brief, 200 mg of DMSNs or DMSNs-Im was placed in a 10 mL glass vial, and 3 mL of FEO was added. After sonication for 5 min to ensure uniform dispersion, the mixture was stirred at 1200 rpm in the dark for 24 h. The resulting product was collected by centrifugation at 12,000 rpm for 20 min, and the precipitate was vacuum-dried at room temperature for 2.5 h, yielding DMSNs-FEO and DMSNs-Im-FEO, respectively. The final products were stored in sealed containers at room temperature until further use. Thermogravimetric analysis (TGA; STA449C, Netzsch, Selb, Germany) was performed on DMSNs, FEO, Im, DMSNs-FEO, and DMSNs-Im-FEO. Samples were heated from room temperature to 600 °C at a heating rate of 10 °C/min under an N_2_ atmosphere at a flow rate of 50 mL/min.

### 2.5. Contact Angle Measurements

The wettability of the DMSNs-Im-FEO formulation on citrus leaf surfaces was evaluated by contact angle measurements. Briefly, 5 µL droplets of an aqueous dispersion of DMSNs-Im-FEO (1 mg mL^−1^) or ultrapure water (control) were deposited onto the adaxial surface of mature lemon leaves. The contact angles were recorded at 5 s post-deposition using a contact angle goniometer [40] (DSA100, KRÜSS, Hamburg, Germany).

### 2.6. Release Behavior of the Formulations

The sustained-release profile of FEO from the DMSNs-Im-FEO formulation was investigated using a gravimetric method [41,42]. Briefly, 150 mg of free FEO and DMSNs-Im-FEO containing an equivalent amount of FEO were placed in quartz evaporating dishes, labeled, and weighed to record the initial total mass (m_0_). The samples were subsequently incubated at 35 °C and weighed at predetermined time intervals (0.5, 1, 2, 5, 24, 72, and 168 h) to determine the remaining total mass (m_t_). The initial FEO mass in DMSNs-Im-FEO (m_FEO,0) was calculated from the FEO loading capacity determined by TGA (32.98%). Since DMSNs and Im are non-volatile under the experimental conditions, the mass loss (m_0_ − m_t_) was attributed to FEO volatilization. The cumulative FEO release was calculated as FEO release (%) = (m_0_ − m_t_)/m_FEO,0 × 100%. Each treatment was performed in triplicate.

The sustained-release behavior of Im from the formulation was evaluated using a dialysis method [24]. An Im methanol solution and DMSNs-Im-FEO dispersed in 5 mL of 30% methanol (aqueous) were separately transferred into dialysis bags with a molecular weight cutoff of 3.5 kDa (Yuanye, Shanghai, China). Each dialysis bag was then immersed in 100 mL of 30% methanol and stirred continuously for 8 h. At designated time intervals (5 min, 15 min, 30 min, 1 h, 2 h, 4 h, and 8 h), 5 mL aliquots of the external dialysate were withdrawn, and an equal volume of fresh 30% methanol was replenished to maintain a constant volume. The Im concentration in the withdrawn samples was determined by UV spectrophotometry at OD_270_ (Text S1 and Figure S1, Supporting Information). All experiments were conducted in triplicate.

### 2.7. UV Stability of Im

The photostability of Im within the DMSNs-Im-FEO formulation was evaluated under UVB irradiation, following previously reported protocols [43]. Briefly, 2 mL of an Im methanol solution (1 g L^−1^) and 2 mL of an aqueous dispersion of DMSNs-Im-FEO (containing an equivalent Im concentration of 1 g L^−1^) were separately dropped onto quartz evaporating dishes and allowed to air-dry. The dried samples were subsequently exposed to UVB irradiation (311 nm, Philips, Amsterdam, The Netherlands, 9 W) at a distance of 10 cm for 8 h or 72 h. After irradiation, the residues were reconstituted in 20 mL of methanol, ultrasonicated for 15 min, and centrifuged. The resulting supernatant was collected, and its absorbance was measured at OD_269_. The residual Im content was calculated as a percentage of the corresponding unirradiated sample at 0 h. Free Im and DMSNs-Im-FEO samples without UV irradiation were used as the 100% reference for their respective irradiated counterparts. Each treatment was performed in triplicate.

### 2.8. Repellent Effect of DMSNs-Im-FEO Against D. citri

We first used a T-tube olfactometer to test the repellent levels of FEO and DMSNs-Im-FEO against *D. citri* (Text S2, Supporting Information). The repellent activity of DMSNs-Im-FEO against *D. citri* on plant surfaces was evaluated using *M. paniculata* plants [29]. Given that FEO exhibits phytotoxicity to citrus leaves (as detailed in the biosafety assessment section) and that DMSNs-Im alone does not possess repellent activity against *D. citri*, only the DMSNs-Im-FEO formulation was tested for its repellent efficacy. Specifically, DMSNs-Im-FEO was sprayed onto *M. paniculata* plants. The treated plants were then placed in mesh cages (100 × 100 × 100 cm). A total of 100 adult *D. citri* individuals (regardless of sex) were released at the center of each cage. Individuals that did not settle on either treated or control plants within 24 h were recorded as non-responders.

### 2.9. Control Efficacy of DMSNs-Im-FEO Against D. citri

A 50 mg mL^−1^ Im stock solution was prepared in acetone under sonication. An aliquot of 1 mL of the Im stock solution was transferred to an evaporating dish, and the acetone was allowed to evaporate completely. The dried Im residue and DMSNs-Im-FEO containing an equivalent amount of Im were separately subjected to UV irradiation for 72 h. Following irradiation, the Im residue was redissolved in 1 mL of acetone. Both the Im and DMSNs-Im-FEO samples were then diluted with deionized water containing 0.1% (*v*/*v*) Triton X-100 to a final Im concentration of 35 mg L^−1^, to facilitate droplet adhesion on the leaf surface. This concentration was selected based on the previously reported bioassay protocol [44]. It is also within the same order of magnitude as typical field application rates for imidacloprid on citrus (50–100 mg/L active ingredient). *M. paniculata* seedlings were sprayed with the respective test solutions until runoff using a hand-held sprayer and then allowed to air-dry. Subsequently, 20 adult *D. citri* individuals were introduced onto each treated seedling, and each treatment was replicated three times. The seedlings were maintained in a controlled environment, and mortality was recorded every 2 days over a 6-day observation period. Insects were considered dead if they remained recumbent and showed no response upon gentle prodding. Control treatments, including acetone-only and DMSNs-FEO, were included for comparison.

### 2.10. Preliminary Non-Target Tolerance Assessment of DMSNs-Im-FEO

The preliminary non-target tolerance assessment of the DMSNs-Im-FEO formulation on citrus leaves was evaluated by assessing the phytotoxicity of FEO and DMSNs-Im-FEO on lemon leaf surfaces, following previously reported methods with appropriate modifications [45]. Briefly, 5 µL droplets of DMSNs-Im-FEO (2 mg mL^−1^), FEO, or ultrapure water (control) were applied onto the adaxial surface of mature lemon leaves, with three replicate applications per treatment. After incubation at room temperature for 7 days, the phytotoxic effects on the leaf surface were visually assessed. The leaves were dark-adapted for 30 min, and leaf photosynthetic parameters were acquired using a large multispectral fluorescence imaging system (PlantExplorer; PhenoVation B.V., Wageningen, The Netherlands). Specifically, the leaves were placed adaxial side up on the imaging platform, the focus was adjusted, and parameters including the maximum photosynthetic efficiency (Fv/Fm), chlorophyll, anthocyanin reflectance index (ARI), and normalized difference vegetation index (NDVI) were obtained by running the built-in program. After data acquisition, the data were imported into Data Analysis (5.6.9) software for analysis, and the exported images were used for subsequent analysis.

The effect of DMSNs-Im-FEO on the germination of green manure seeds was further examined [40]. Seeds of *A. sinicus* were surface-sterilized with 5% sodium hypochlorite solution for 20 min and subsequently rinsed thoroughly with ultrapure water. The sterilized seeds (50 seeds per dish) were then placed in 9 cm Petri dishes lined with filter paper, to which 2 mL of DMSNs-Im-FEO aqueous dispersions at concentrations of 0.5, 1.0, and 2.0 mg mL^−1^ were added. Each treatment was performed in triplicate. The dishes were covered with autoclaved moist tissue paper to maintain humidity and incubated at 25 °C for 5 days. Seeds were considered germinated when the radicle protruded through the seed coat by at least 2 mm [46]. Germination and seedling length were recorded after 5 days of incubation.

The impact of DMSNs-Im-FEO on earthworm survival was analyzed following previously established protocols [47]. Specifically, DMSNs-Im-FEO aqueous dispersions were incorporated into 50 g of artificial soil to achieve final concentrations of 10, 20, 40, and 80 mg kg^−1^. The soil was placed in breathable containers, and 20 earthworms (*Eisenia fetida*) were introduced into each container. The containers were maintained in a humidified environment at 26 °C in the dark. After 7 days of exposure, earthworm mortality was recorded.

### 2.11. Statistical Analysis

All data are presented as mean ± standard deviation (SD). For comparisons between two groups, Student’s t-test was used. For comparisons among multiple groups, one-way analysis of variance (ANOVA) followed by Tukey’s post hoc test was employed. A *p*-value < 0.05 was considered statistically significant. All statistical analyses were performed using GraphPad Prism (v9, GraphPad Software, Boston, MA, USA). Figures were generated using Origin 2024 (v2024, OriginLab Corp., Northampton, MA, USA).

## 3. Results and Discussion

### 3.1. Synthesis and Characterization of DMSNs

The construction of the DMSNs-Im-FEO system is illustrated in Scheme 1. DMSNs were prepared via the sol-gel process in the presence of CTAB, NaSal, and TEA. The average particle size of DMSNs was approximately 257 nm (Figure 1a and Figure S2), which is consistent with previously reported values [37]. Notably, the adsorption of Im and FEO did not significantly alter the structural integrity of the DMSNs; however, evident payloads were observed within the mesopores (Figure 1b), providing preliminary evidence for the successful loading of both active ingredients into the DMSNs channels.

The textural properties of the materials were then characterized by N_2_ adsorption–desorption measurements (Figure 2a–c). DMSNs exhibited a high specific surface area (424.32 m^2^ g^−1^) and a large total pore volume (2.031 cm^3^ g^−1^), indicating a well-developed mesoporous structure. After Im loading, however, both the specific surface area and total pore volume decreased markedly to 128.67 m^2^ g^−1^ and 0.742 cm^3^ g^−1^, respectively, suggesting that Im entered and occupied part of the mesoporous channels. Upon further introduction of FEO, the nitrogen-accessible specific surface area and pore volume almost completely disappeared (2.97 m^2^ g^−1^ and 0.00993 cm^3^ g^−1^, respectively). This indicates that FEO further filled the pores and possibly covered or blocked the pore openings, thereby confirming that Im and FEO had occupied the interior of the mesopores. Consistent with this interpretation, the pore size distribution showed a distinct peak at 26.08 nm for DMSNs, a decreased peak intensity at 27.89 nm for DMSNs-Im, and no well-defined mesoporous peak for DMSNs-Im-FEO. Taken together, these results suggest that the pore channels were progressively occupied during the loading process.

The loading capacities of each component were further quantified by thermogravimetric analysis (Figure 2b,c). In the single-adsorption system, the loading efficiencies of DMSNs for FEO and Im were 30.39% and 44.81%, respectively (the latter being close to the value of 46.24% determined by UV spectrophotometry, Figure S1). In the DMSNs-Im-FEO co-loading system, the loading rates of FEO and Im were 32.98% and 21.79%, respectively, indicating a decrease in the adsorption efficiency for Im in the competitive loading system. The decrease in Im loading in the co-loaded system (21.79%) compared with the single-loaded system (44.81%) is likely attributable to competitive adsorption and partial elution during the subsequent FEO loading step. Since the mesoporous channels and adsorption sites of DMSNs are limited, FEO molecules compete with the pre-loaded Im for pore space and binding sites. In addition, FEO, as a hydrophobic essential oil, may partially dissolve or displace Im from the DMSNs during the second loading step, resulting in a lower final Im loading capacity. Nevertheless, DMSNs successfully achieved the co-loading of FEO and Im.

Further confirmation of the loading process was obtained via FT-IR spectroscopy (Figure 2d). Compared with the spectrum of DMSNs, that of DMSNs-Im exhibited characteristic absorption bands at 3354 cm^−1^ (N–H stretching), 1732 cm^−1^ (C=O carbonyl stretching), and 1298 cm^−1^ (C–N stretching), along with peaks at 1563 cm^−1^ (C=C skeletal vibration) and 744 cm^−1^ (aromatic C–H out-of-plane bending) [48]. These features are assignable to Im, while the Si–O–Si framework peak at 468 cm^−1^ remained intact [49], confirming the successful loading of Im without disruption to the mesoporous structure. In the DMSNs-Im-FEO spectrum, the C–H stretching (2957 cm^−1^) and bending (1435 cm^−1^) bands—originating from alkanes and terpenes in FEO—were markedly enhanced, and the C=C peak at 1563 cm^−1^ also exhibited a clear increase, consistent with the olefinic characteristics of the essential oil [50]. Importantly, the Im-related peaks (N–H at 3354 cm^−1^, C=O at 1732 cm^−1^, and C–N at 1298 cm^−1^) remained clearly identifiable in the co-loaded system, indicating that Im was neither displaced nor degraded during the subsequent FEO loading step. Collectively, these results demonstrate that Im was first stably immobilized within the DMSNs matrix, followed by the successful entry of FEO into the same carrier, thereby achieving co-loading of both agents in a single nanoplatform. It should be noted that the FEO loading capacity was determined by TGA as a semi-quantitative reference. Because FEO is a multi-component essential oil, its precise independent quantification would require GC or GC-MS analysis, which was not performed in this study. Therefore, the absolute FEO loading value should be regarded as semi-quantitative, whereas the relative loading trends of Im and FEO are still supported by the current data.

### 3.2. Sustained-Release Performance of DMSNs-Im-FEO

The sustained release of active ingredients is critical for prolonging their field efficacy. In this study, upon exposure to the release medium, the release of Im from DMSNs-Im-FEO was slower than that of free Im (Figure 3a). This retardation is likely attributable to the mesoporous architecture of DMSNs, which physically impedes the access of the release medium to the encapsulated Im, thereby achieving a typical sustained-release profile [21]. Such a release behavior is beneficial for enhancing the long-term control of target pests [18]. The release medium (30% methanol) was selected because imidacloprid has limited solubility in pure water, which prevents the establishment of a measurable release gradient. This medium allows the relative release behavior of free Im and DMSNs-Im-FEO to be compared under identical conditions. However, we acknowledge that this system does not fully represent field conditions, and future field studies will be needed to validate the actual release behavior on plant surfaces. In addition, FEO was confirmed to have little interference with the UV-Vis determination of Im in this release assay, as the dialysate of DMSNs-FEO showed weak absorbance without an obvious characteristic absorption peak at 270 nm, which was much lower than that of the DMSNs-Im-FEO dialysate (Figure S3).

Plant essential oils such as FEO exhibit notable advantages in antibacterial activity and repellency against *D. citri* (Figure S4); however, their high volatility substantially restricts their practical deployment [29,32]. In the present work, FEO was successfully encapsulated within the dendritic mesopores of DMSNs, and a slower release rate was observed compared with that of free FEO (Figure 3b). This indicates that DMSNs not only serve as an effective carrier for FEO loading but also function as a favorable matrix for achieving its sustained release. Collectively, these findings demonstrate that in the DMSNs-based co-loading system, both Im and FEO exhibit sustained-release properties, thereby laying a solid foundation for further evaluation of the formulation under field conditions.

### 3.3. UV Stability of DMSNs-Im-FEO

Poor photostability is a major factor contributing to the shortened field persistence of pesticides such as Im [39]. In this study, after exposure to UVB irradiation for 8 h and 72 h, the residual content of free Im decreased to 69.68% and 38.31% of the initial amount, respectively. By contrast, the DMSNs-Im-FEO group retained 90.66% and 72.79% of the initial Im content under identical irradiation conditions (Figure 4), indicating that DMSNs confer substantial photoprotection to Im, which may contribute to prolonged efficacy under field conditions; however, this remains to be validated in field trials. This protective effect is likely attributable to the ability of DMSNs to reflect or scatter UV radiation, thereby reducing the UV energy reaching the encapsulated pesticide molecules and acting as an effective physical shielding barrier [20,22]. The present results demonstrate that encapsulation within DMSNs enhances the photostability of Im under UVB irradiation relative to free Im. However, because DMSNs-Im and DMSNs-FEO controls were not included, the respective contributions of the carrier and FEO to this protection cannot be separated in this study. Future studies will include these controls to systematically evaluate the individual roles of the carrier and FEO.

### 3.4. Control Efficacy of DMSNs-Im-FEO Against D. citri

The wettability of the DMSNs-Im-FEO formulation on citrus leaf surfaces was first evaluated. Compared with water, the aqueous dispersion of DMSNs-Im-FEO exhibited a lower contact angle on citrus leaves (Figure S5), indicating improved wettability [39], which is beneficial for facilitating subsequent field application.

Subsequently, a T-tube olfactometer was used to test the repellent levels of FEO and DMSNs-Im-FEO against *D. citri*. Both FEO and DMSNs-Im-FEO were found to possess significant repellent activity against *D. citri* (Figure 5a,b and Figure S3, *p* < 0.01). Given that direct application of FEO to citrus leaves tends to induce phytotoxicity (as discussed in Section 3.5), we directly assessed the repellent activity of DMSNs-Im-FEO against *D. citri* in mesh-cage assays. As a result, *D. citri* adults preferentially avoided *M. paniculata* plants sprayed with DMSNs-Im-FEO, with the majority settling on untreated plants (Figure 5c,d). In further observations, we found that not all *D. citri* individuals within the cage flew toward the *M. paniculata* plants, a phenomenon consistent with previous reports [29]. We speculate that this may be due to the dispersal of FEO released from DMSNs-Im-FEO throughout the confined cage space, which may have driven *D. citri* to move toward the upper parts of the cage. Overall, these results demonstrate that DMSNs-Im-FEO successfully achieved repellent activity against *D. citri*.

Furthermore, we compared the insecticidal efficacy of free Im and DMSNs-Im-FEO against *D. citri* following 72 h of UV pre-exposure. No mortality was observed in any treatment group at 24 h post-treatment; however, UV-stressed DMSNs-Im-FEO exhibited significantly higher insecticidal activity than UV-stressed free Im at both 2 and 4 days post-treatment (Figure 6), which is consistent with the photoprotection results presented in Figure 4. This confirms that encapsulation within DMSNs confers effective protection against UV-induced degradation. Collectively, the repellency and insecticidal bioassay results demonstrate that DMSNs not only extend the persistence of the active ingredients but also improve the overall control efficacy against *D. citri* through sustained co-delivery. Nevertheless, these results were obtained under laboratory conditions, whereas pesticide application in the field is inherently complex. Drawing on previous experience with precision-engineered polymer nanostructures [51], rational tailoring of DMSNs morphology, size, and surface chemistry may further enhance drug loading and target-responsive release [24], thereby improving field control efficacy against plant diseases and pests.

### 3.5. Preliminary Non-Target Tolerance Assessment of DMSNs-Im-FEO

Preliminary non-target tolerance is an important prerequisite for assessing the field application potential of pesticide formulations. In this study, we preliminarily evaluated the biological toxicity of DMSNs-Im-FEO toward citrus plants, Chinese milk vetch, and earthworms. Results from the citrus leaf assay showed that direct application of FEO caused pronounced phytotoxicity, including tissue damage, cell death at the application site, and leaf curling. By contrast, DMSNs-Im-FEO applied to the leaf surface did not induce any visible damage to citrus leaf tissues (Figure 7a). Representative false-color images of leaf photosynthetic parameters also showed that FEO treatment altered the photosynthetic performance of citrus leaves, whereas DMSNs-Im-FEO treatment showed no obvious changes compared with the control (Figure 7b). The cuticular wax layer serves as a key protective barrier against environmental stresses in citrus and other plants [52]. When lipophilic FEO components (mainly comprising 2-*β*-pinene, *α*-pinene, *δ*-3-carene, and camphene [29]) come into contact with this layer, they can disrupt the wax structure [53], thereby inducing phytotoxicity. However, FEO encapsulated within the mesopores of DMSNs is prevented from making direct contact with the leaf surface, thus avoiding damage to leaf tissues. Furthermore, we assessed the effect of DMSNs-Im-FEO on the germination of *A. sinicus* seeds. The results showed that DMSNs-Im-FEO had no significant effects on seed germination rate and seedlings length (Figure 7c–e, *p* > 0.05), further supporting its preliminary non-target tolerance. In addition, we evaluated the toxicity of the formulation to earthworms in soil. The results also indicated that soil exposure to DMSNs-Im-FEO did not cause any obvious adverse effects on earthworm survival (Figure 7f, *p* > 0.05). Collectively, these findings suggest that DMSNs-Im-FEO shows favorable preliminary non-target tolerance and has potential for the sustainable management of *D. citri*.

## 4. Conclusions

In this study, we developed a DMSNs-based co-delivery formulation loaded with Im and FEO. SEM and TEM observations, together with BET, TGA, and FT-IR analyses, support the successful loading of both active ingredients into the DMSNs. The formulation exhibited sustained release of Im and FEO under the tested conditions and improved the photostability of Im under UVB irradiation relative to free Im. Furthermore, the encapsulation and physical barrier effect of DMSNs substantially alleviated the phytotoxicity of FEO to citrus leaves and showed favorable preliminary non-target tolerance to the DMSNs-Im-FEO formulation toward *A. sinicus* and earthworms. Collectively, our findings provide a long-lasting strategy for the sustainable management of *D. citri*, which holds significant implications for the integrated control of HLB and the sustainable development of the global citrus industry. However, the present efficacy and non-target safety data were obtained under laboratory conditions only. Field efficacy trials and longer-term, multi-species non-target safety assessments remain necessary in future studies.

## Figures and Tables

**Scheme 1:**
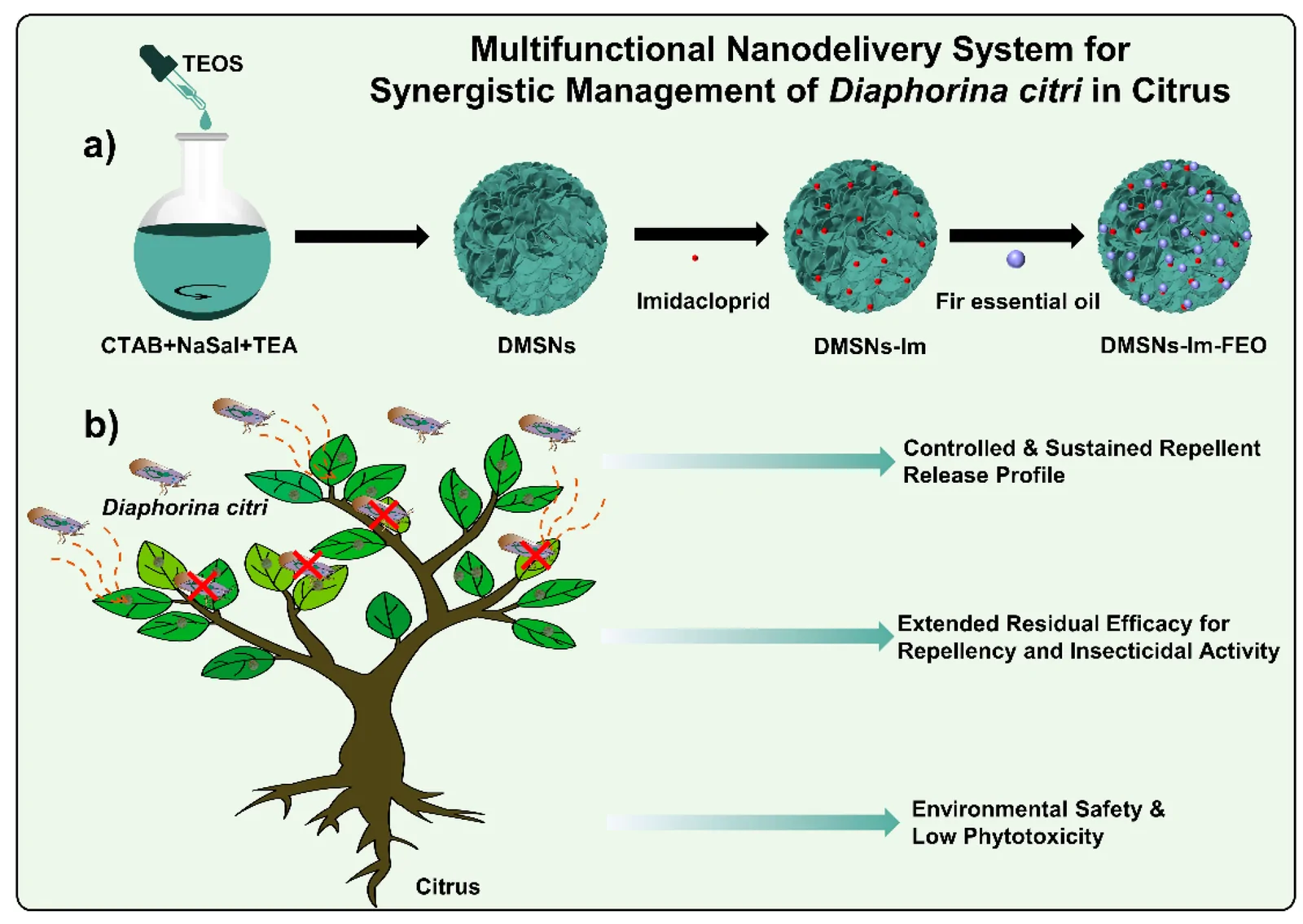
Schematic illustration of the fabrication and dual-functional action of DMSNs-Im-FEO for sustained control of *D. citri*. (**a**) Preparation process of the DMSNs-Im-FEO nanoformulation. (**b**) Proposed modes of action of DMSNs-Im-FEO against *D. citri*. Dashed lines indicate FEO volatilization and repellency; cross symbols indicate *D. citri* mortality.

**Figure 1:**
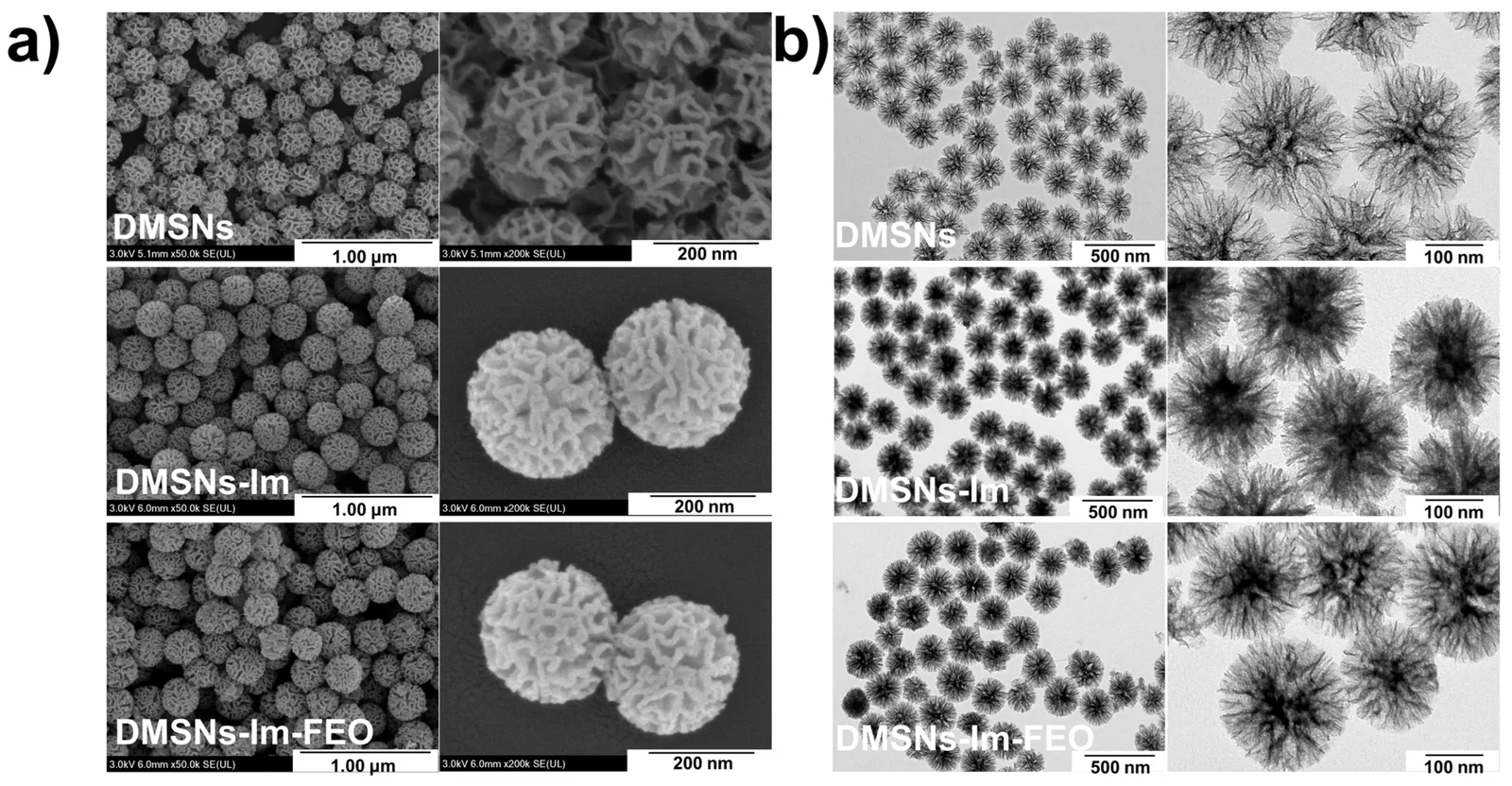
(**a**) SEM and (**b**) TEM images of DMSNs before and after loading with Im and FEO. Scale bars are indicated in the images.

**Figure 2:**
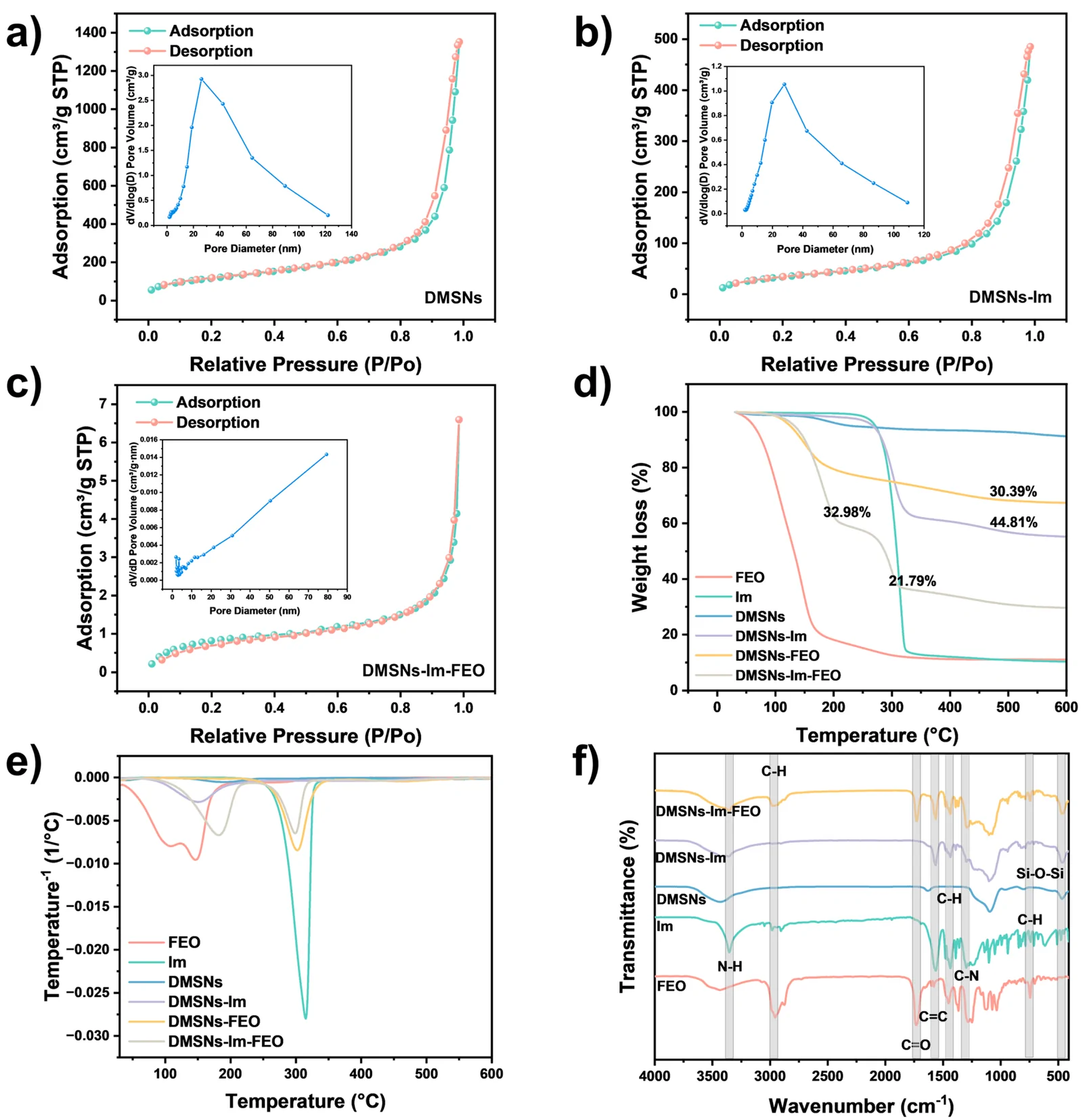
(**a**) N_2_ adsorption–desorption isotherms and pore size distribution (inset) of DMSNs. (**b**) N_2_ adsorption–desorption isotherms and pore size distribution (inset) of DMSNs-Im. (**c**) N_2_ adsorption–desorption isotherms and pore size distribution (inset) of DMSNs-Im-FEO. (**d**) TGA and (**e**) DTG curves of FEO, Im, DMSNs, DMSNs-Im, and DMSNs-Im-FEO. (**f**) FT-IR spectra of FEO, Im, DMSNs, DMSNs-Im, and DMSNs-Im-FEO over the wavenumber range of 4000–400 cm^−1^. Characteristic absorption bands are labeled, including Si–O–Si (468 cm^−1^), C–H (2957, 1435, and 744 cm^−1^), C=O (1732 cm^−1^), C=C (1563 cm^−1^), C–N (1298 cm^−1^), and N–H (3354 cm^−1^).

**Figure 3:**
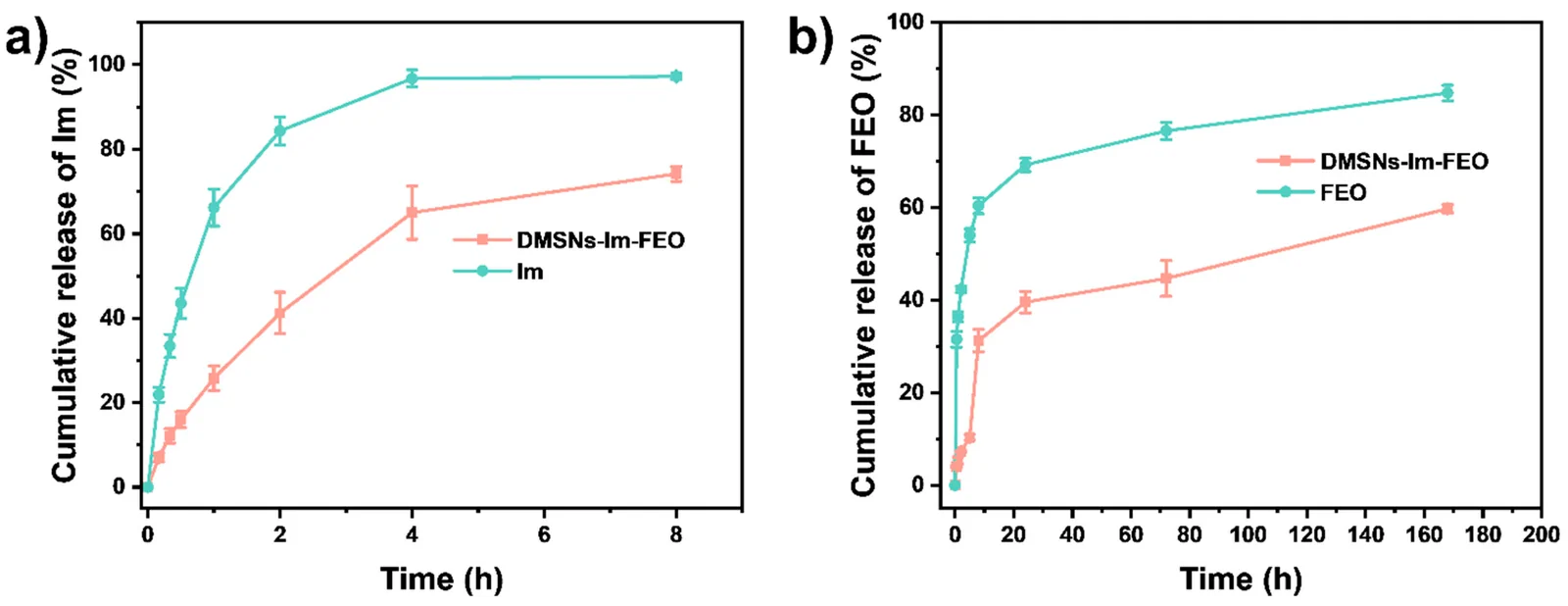
Sustained-release profiles of Im and FEO from DMSNs-Im-FEO. (**a**) Cumulative release of free Im versus Im from DMSNs-Im-FEO over time. (**b**) Cumulative release of free FEO versus FEO from DMSNs-Im-FEO over time. All data points are presented as mean ± SD from three independent experiments (n = 3).

**Figure 4:**
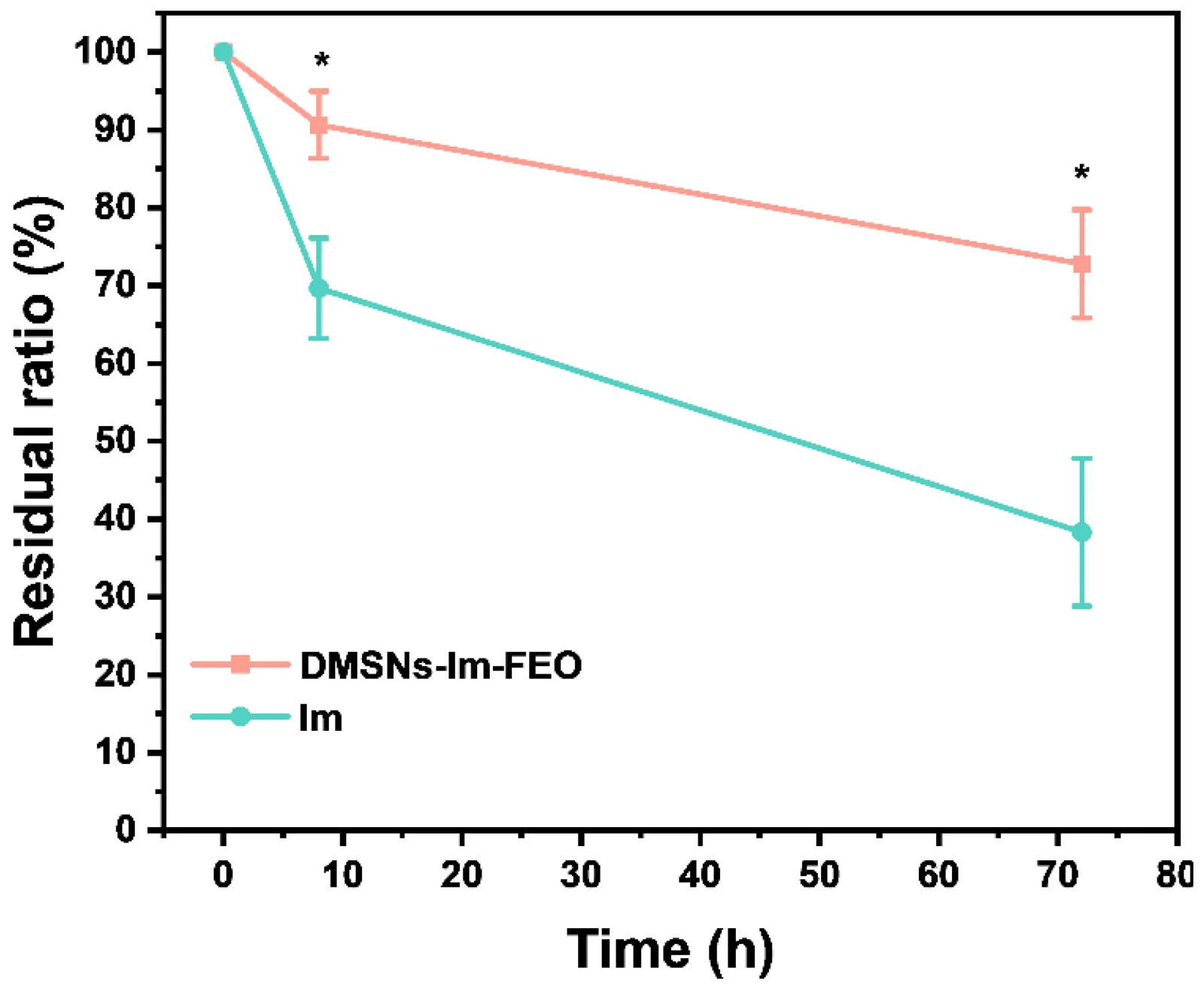
The UV degradation curves of Im and DMSNs-Im-FEO. * indicates a significant difference (*p* < 0.05) between the two treatments.

**Figure 5:**
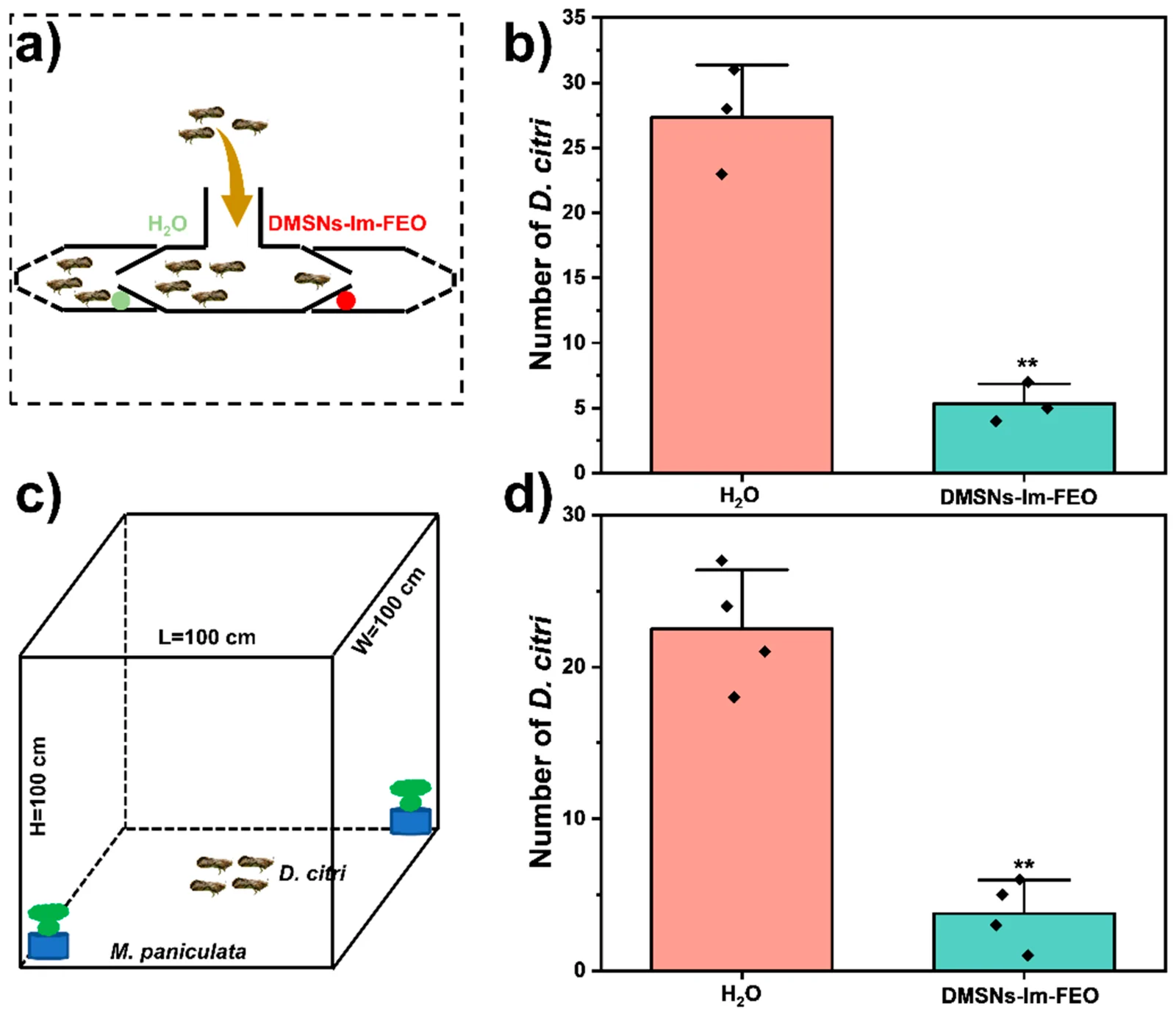
Repellent activity of DMSNs-Im-FEO against *D. citri*. (**a**) Schematic diagram of the T-tube olfactometer. (**b**) Statistical summary of the results from three independent replicates (n = 3 cages). (**c**) Schematic diagram of the repellency test with *M. paniculata*. (**d**) Number of *D. citri* settling per plant on *M. paniculata* treated with DMSNs-Im-FEO after 24 h compared with H_2_O in a choice bioassay. Data are presented as mean ± SD (n = 3 independent cages). ** *p* < 0.01.

**Figure 6:**
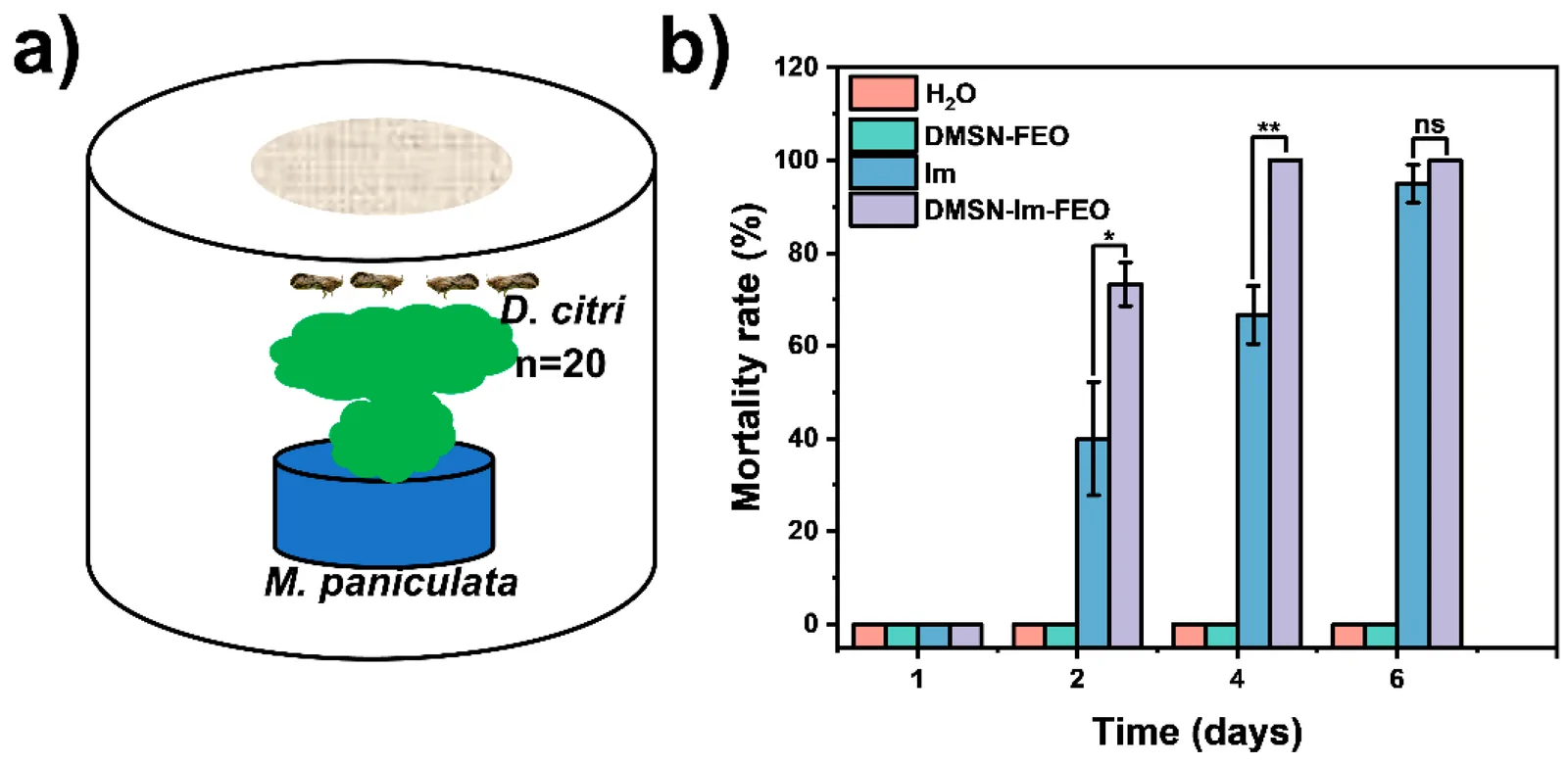
Effects of UV irradiation on DMSNs-Im-FEO and Im for the control of *D. citri*. (**a**) Schematic diagram of bioassay for *D. citri*. (**b**) Bioassay results of *D. citri* samples at different treatment time points. * indicates a significant difference (*p* < 0.05) between the two treatments. ** indicates a highly significant difference between the two treatments at *p* < 0.01. ns indicates no significant difference between the two treatments at *p* > 0.05.

**Figure 7:**
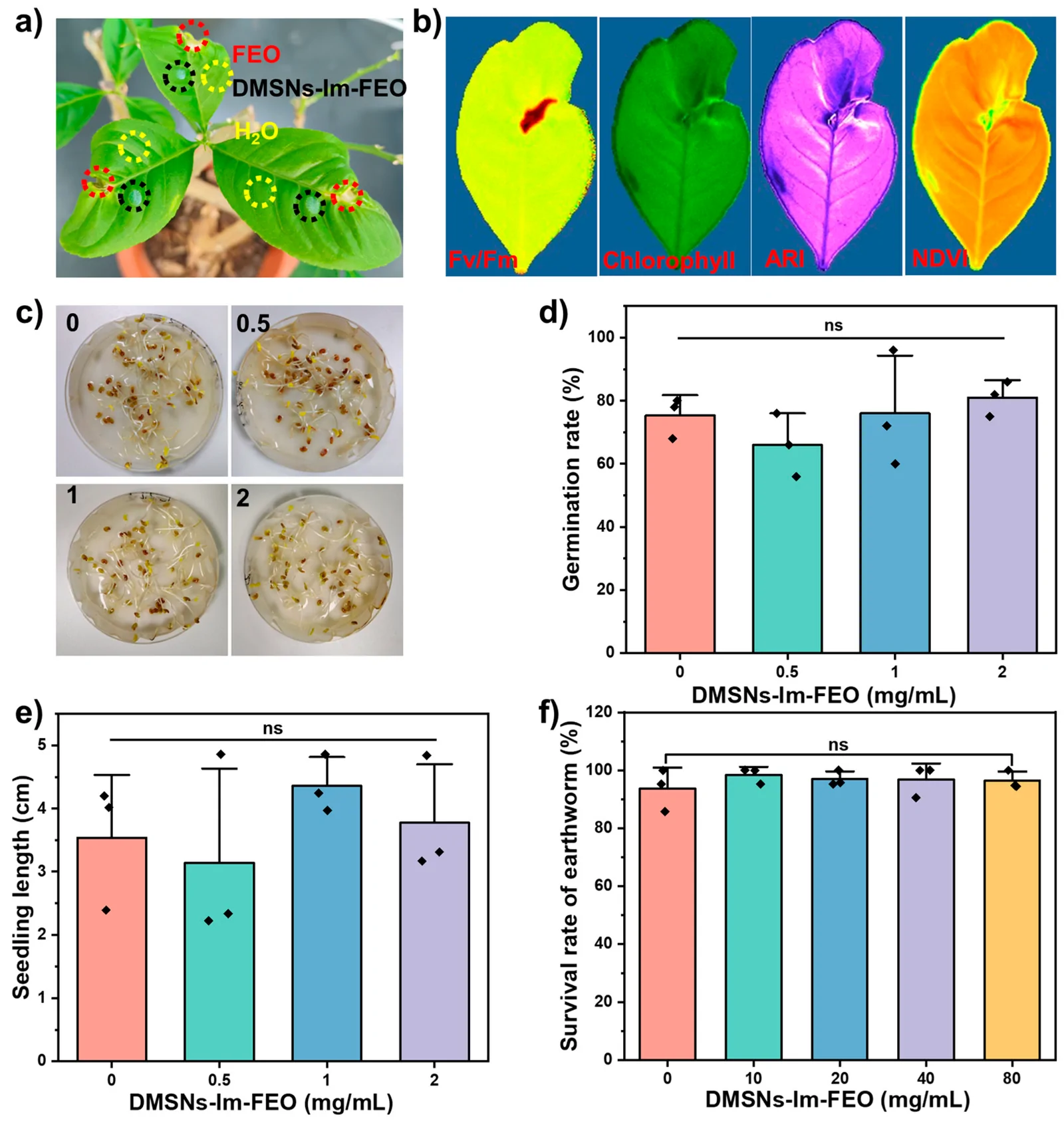
Preliminary non-target tolerance assessment of DMSNs-Im-FEO. (**a**,**b**) Effect of FEO and DMSNs-Im-FEO on citrus leaves. 0 represents the untreated control (water-sprayed). Fv/fm for maximum photosynthetic efficiency; Chlorophyll for all chlorophylls; anthocyanin reflectance index (ARI) for anthocyanin reflectance index; and normalized difference vegetation index (NDVI) for normalized vegetation index. (**c**–**e**) The effects of DMSNs-Im-FEO on *A. sinicus* seed germination and seedlings length. (**f**) Survival rate of earthworms after 7 days of exposure to DMSNs-Im-FEO. Data are presented as mean ± SD. ‘ns’ indicates no significant difference (*p* > 0.05).

## Data Availability

The original contributions presented in this study are included in the article/Supplementary Material. Further inquiries can be directed to the corresponding authors.

## Supplementary Materials

The following supporting information can be downloaded at: https://www.mdpi.com/article/10.3390/nano16191206/s1, Text S1. Imidacloprid Loading Capacity of DMSNs; Text S2. Evaluation of the Repellent Activity of FEO Against *D. citri.* Figure S1: UV–Vis absorption spectra and standard calibration curves of imidacloprid (Im). (a) Absorption spectra of Im at various concentrations in 100% methanol. (b) Corresponding linear calibration curve at the maximum absorption wavelength of 269 nm. (c) Absorption spectra of Im at various concentrations in 30% methanol. (d) Corresponding linear calibration curve at the maximum absorption wavelength of 270 nm; Figure S2: Particle size distribution of DMSNs determined from SEM images using Nano Measurer 1.2 software. Particle diameters are presented as mean ± SD based on measurements of 200 individual nanoparticles from SEM micrographs. The Gaussian fitting curves are included solely for the purpose of visualizing the particle size distribution; Figure S3. UV–Vis absorption spectra of the dialysate of DMSNs-FEO. DMSNs-FEO (150 mg, the same amount as DMSNs-Im-FEO used in the Im release experiment) was dialyzed, and the dialysate was diluted using the same dilution ratio as that used in the Im release experiment. Figure S4: Evaluation of the repellent activity of FEO against *D. citri*. (a) Schematic diagram of the T-tube olfactometer used in the bioassay. (b) Representative images showing the repellent response of *D. citri* to FEO. (c) Statistical summary of the results from three independent replicates. ** Indicates a highly significant difference between the two treatments at *p* < 0.01; Figure S5: The contact angle of different solutions (water and DMSNs-Im-FEO) on the surfaces of lemon leaves at 5 s.

## Abbreviations

The following abbreviations are used in this manuscript:

HLB	Huanglongbing
*D. citri*	*Diaphorina citri*
*C*las	*C*andidatus Liberibacter asiaticus
Im	Imidacloprid
FEO	Fir essential oil
DMSNs	Dendritic mesoporous silica nanoparticles
MSNs	Mesoporous silica nanoparticles
CTAB	Cetyltrimethylammonium bromide
TEOS	Tetraethyl orthosilicate
NaSal	Sodium salicylate
TEA	Triethanolamine
HCl	Hydrochloric acid
SEM	Scanning electron microscopy
TEM	Transmission electron microscopy
FT-IR	Fourier-transform infrared spectroscopy
TGA	Thermogravimetric analysis
DTG	Derivative thermogravimetry
BET	Brunauer-Emmett-Teller
UV	Ultraviolet
UVB	Ultraviolet B
OD	Optical density
SD	Standard deviation
*A. sinicus*	*Astragalus sinicus*
*E. fetida*	*Eisenia fetida*
Fv/fm	Maximum photosynthetic efficiency
ARI	Anthocyanin reflectance index
NDVI	Normalized difference vegetation index
Rpm	Revolutions per minute
kDa	Kilodalton
mg mL^−1^	Milligram per milliliter
g L^−1^	Gram per liter
