# Supplementary material for: Dendritic Mesoporous Silica Nanoparticle-Based Co-Delivery of a Repellent and an Insecticide for Management of the Asian Citrus Psyllid
Source: Nanomaterials (Basel). 2026 Sep 24;16(19):1206. doi: 10.3390/nano16191206 (PMC13666863; doi:10.3390/nano16191206)
Supplement: Supplementary file 1 [file nanomaterials-16-01206-s001.zip › nanomaterials-4568996-supplementary.pdf]

Support Information for

Dendritic Mesoporous Silica Nanoparticle-Based Co-Delivery of a Repellent and an Insecticide for Management of the Asian Citrus Psyllid

Wenhua Rao[1], Qianying Li[1, 2], Kaidi Wang[1], Hua Chen[3], Lan Li[4], Chao Shen[1], Guocheng Fan[1*], and Jinfeng Hu[1*]

1 Fujian Engineering Research Center for Green Pest Management, Fujian Key Laboratory for Monitoring and Integrated Management of Crop Pests, Fuzhou Scientific Observing and Experimental Station of Crop Pests of Ministry of Agriculture and Rural Affairs, Institute of Plant Protection, Fujian Academy of Agricultural Sciences, Fuzhou, 350013, China

2 College of Plant Protection, Fujian Agriculture and Forestry University, Fuzhou, 350002, China

3 Ningde Inspection and Testing Centre for Agricultural Product Quality and Safety, Ningde, 352100, China

4 Fujian Provincial Center for Agri-product Quality Safety Inspection and Testing, 350003, China

Corresponding Authors

*Tel. & Fax: +086-0591-87810769. E-mail: guochengfan@126.com (Guocheng Fan)

*Tel. & Fax: +086-0591-87810769. E-mail: hujinfeng007@sina.com (Jinfeng Hu)

Text S1. Imidacloprid Loading Capacity of DMSNs

To construct the standard calibration curve for Im, a stock solution of 1 mg mL^{-1} was prepared in methanol and subsequently diluted with the same solvent to obtain a series of concentrations ranging from 0.001 to 0.030 mg mL^{-1} (0.001, 0.005, 0.010, 0.015, 0.020, 0.025, and 0.030 mg mL^{-1}). The UV–Vis absorption spectra of these solutions were recorded over the wavelength range of 200–350 nm using a UV–Vis spectrophotometer (T10, Purkinje), with pure methanol serving as the blank. The absorbance values at 269 nm were then measured for each concentration. A standard curve was established by linear regression analysis, with a correlation coefficient (R^2) exceeding 0.99 in all cases.

Separately, an additional calibration curve was constructed using 30% methanol as the solvent. A 0.1 mg mL^{-1} stock solution of Im was prepared in 30% methanol and diluted with the same solvent to the identical concentration range (0.001–0.030 mg mL^{-1}). The UV–Vis absorption spectra were scanned from 200 to 350 nm against a blank of 30% methanol, and the absorbance at 270 nm was recorded. A linear calibration curve was derived via linear regression, also achieving an R^2 value greater than 0.99.

To evaluate the loading capacity of Im onto DMSNs, 10 mg of Im-loaded DMSNs (DMSNs–Im and DMSNs-Im-FEO) was dispersed in 25 mL of methanol. The mixture was sealed and ultrasonicated for 2 h, with ice cubes added intermittently during the process to maintain the temperature at approximately 30 °C. After ultrasonication, the supernatant was collected, appropriately diluted with methanol, and its absorbance at

269 nm was measured using a spectrophotometer. The amount of Im loaded onto the DMSNs was then calculated based on the pre-established calibration curve.

Text S2. Evaluation of the Repellent Activity of FEO Against *D. citri*

The repellent activity of FEO against *D. citri* was evaluated using a T-tube olfactometer. Fifty adult *D. citri* individuals were introduced into the entrance port of the olfactometer. A circular filter paper disc (diameter ≈ 0.5 cm) was placed at each of the two terminal arms of the olfactometer. One disc was impregnated with 5 μL of distilled water (serving as the control), while the other received 5 μL of FEO or 5 mg DMSNs-Im-FEO (serving as the treatment). The number of *D. citri* individuals present at each terminal end was recorded after 24 h of exposure.

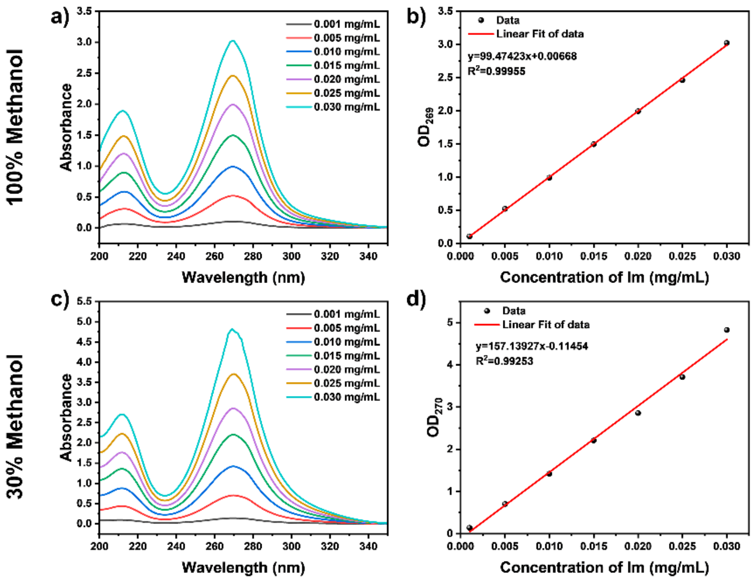

Figure S1. UV–Vis absorption spectra and standard calibration curves of imidacloprid (Im). (a) Absorption spectra of Im at various concentrations in 100% methanol. (b) Corresponding linear calibration curve at the maximum absorption wavelength of 269 nm. (c) Absorption spectra of Im at various concentrations in 30% methanol. (d) Corresponding linear calibration curve at the maximum absorption wavelength of 270 nm.

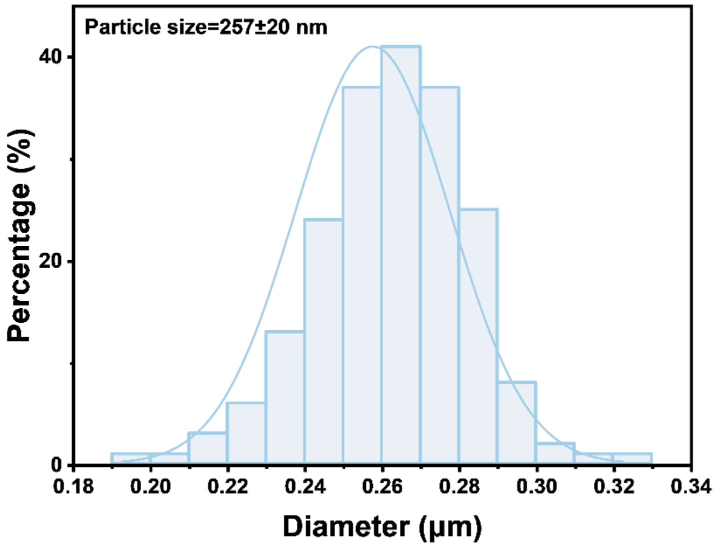

Figure S2. Particle size distribution of DMSNs determined from SEM images using Nano Measurer 1.2 software. Particle diameters are presented as mean ± SD based on measurements of 200 individual nanoparticles from SEM micrographs. The Gaussian fitting curves are included solely for the purpose of visualizing the particle size distribution.

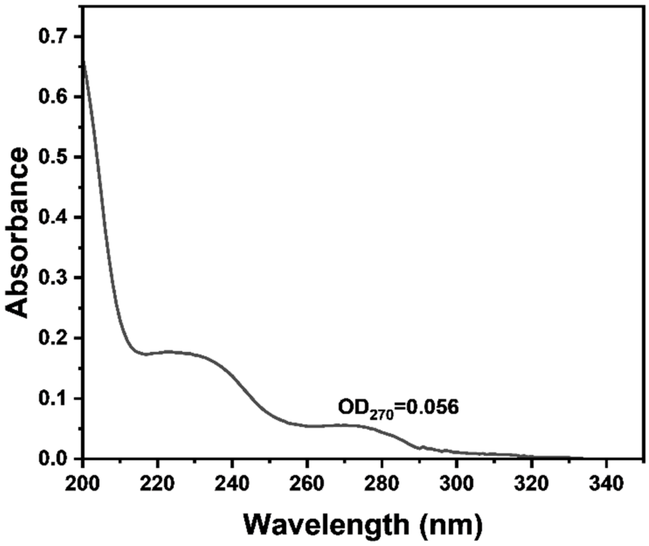

Figure S3. UV–Vis absorption spectra of the dialysate of DMSNs-FEO. DMSNs-FEO (150 mg, the same amount as DMSNs-Im-FEO used in the Im release experiment) was dialyzed, and the dialysate was diluted using the same dilution ratio as that used in the Im release experiment.

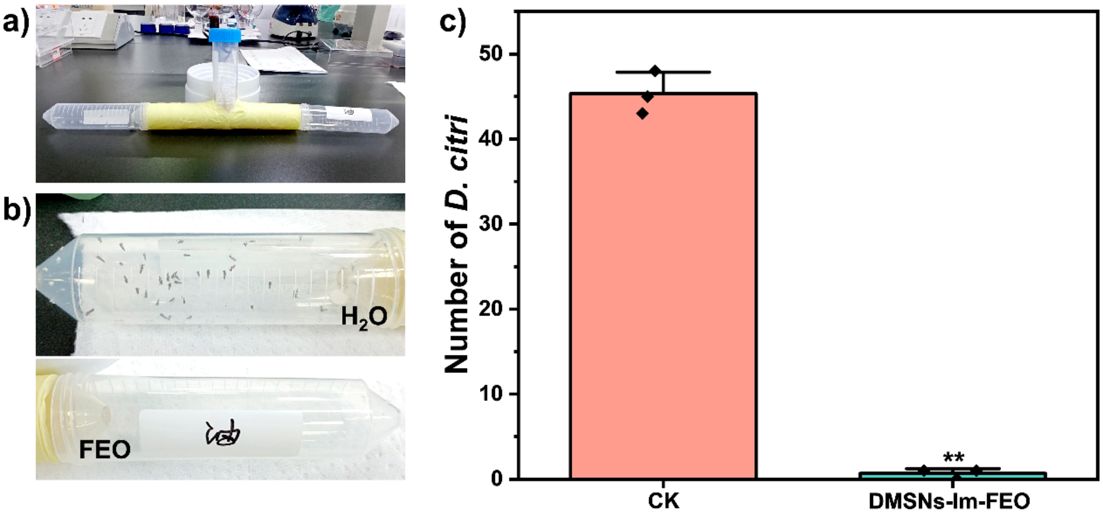

Figure S4. Evaluation of the repellent activity of FEO against *D. citri*. (a) Schematic diagram of the T-tube olfactometer used in the bioassay. (b) Representative images showing the repellent response of *D. citri* to FEO. (c) Statistical summary of the results from three independent replicates. **Indicates a highly significant difference between the two treatments at $P < 0.01$.

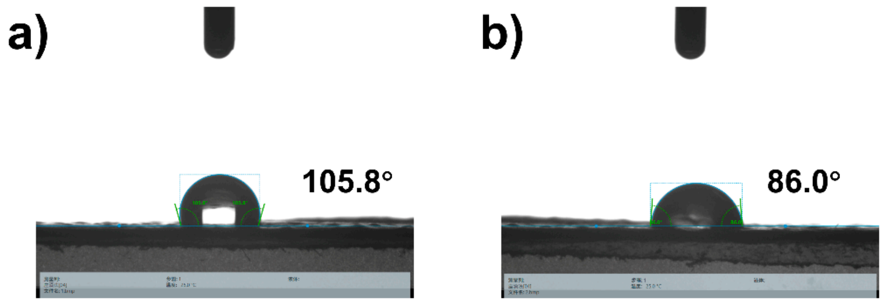

Figure S5. The contact angle of different solutions (water and DMSNs-Im-FEO) on the surfaces of lemon leaves at 5 s.
